# Introgression of thermal tolerance alleles drives adaptation despite risk of mitonuclear conflict

**DOI:** 10.1093/molbev/msag158

**Published:** 2026-07-09

**Authors:** Kamron L Kayhani, Jasmine R Weaver, Molly K Burke, Felipe S Barreto

**Affiliations:** Department of Integrative Biology, Oregon State University, Corvallis, ORUSA; Department of Integrative Biology, Oregon State University, Corvallis, ORUSA; Department of Integrative Biology, Oregon State University, Corvallis, ORUSA; Department of Integrative Biology, Oregon State University, Corvallis, ORUSA

**Keywords:** experimental evolution, cytonuclear coadaptation, *Tigriopus*, hybrids

## Abstract

Hybridization may offer a form of genetic rescue from warming temperatures through the introgression of heat-adapted alleles from a high-tolerance population into a low-tolerance population. However, the success of adaptive introgression can be impeded by incompatibility at other loci, especially those that participate in coadapted gene complexes that could lead to unfavorable combinations in hybrids. We tested the success of introgression of heat-adapted alleles in the face of potential fitness tradeoffs associated with hybridized mitonuclear gene complexes in the crustacean *Tigriopus californicus*. We created reciprocal hybrid crosses of the San Diego (SD) and Strawberry Hill (SH) populations, which show divergence in both thermal tolerance and mitochondrial genomes, then subjected hybrid populations to ten generations of either selection for thermal tolerance or to control conditions. Lines under selection in both crosses evolved higher thermal tolerance. Moreover, both crosses showed substantial increases in nuclear SD allele frequencies, with the SH♀xSD♂ cross having as much SD introgression as the reciprocal cross despite having higher risk of mitonuclear incompatibilities with its SH mtDNA. This suggests that introgression of warm-adapted alleles was largely successful despite the risk of introducing mitonuclear incompatibilities that could result from genome-wide shifts toward the paternal allele. This outcome was likely made possible by strong mitonuclear matching being maintained in a few genomic regions on different chromosomes, exclusive of those responding to thermal selection, pointing to components of cytochrome *c* oxidase, in particular *COX6A1*, as potentially having a disproportionate impact in mitonuclear coadaptation.

## Introduction

Changing thermal environments are intensifying selection for higher levels of thermal tolerance on populations that have previously adapted to local temperature regimes (Sanford and Kelly 2011; Sunday et al. 2015; Smale et al. 2019). One mechanism by which novel beneficial alleles are introduced into a lower-tolerance population is by introgressive hybridization with a more tolerant population (Hedrick 2013; Pereira et al. 2014; Martin et al. 2020; Nanaei et al. 2023). The effectiveness of this adaptive introgression, however, may be impacted by multiple evolutionary processes (Bell 2013; Orr and Unckless 2014; Edelman and Mallet 2021). For instance, introgression of favorable alleles may be prevented or slowed if those alleles are also maladaptive regarding other components of the new environment (Schiffers et al. 2013; Payne et al. 2024), or if they are linked to other loci harboring maladaptive alleles that experience stronger negative selection, especially when recombination or the number of generations is low (Sachdeva and Barton 2018). Adaptive introgression in hybridizing populations is thus susceptible to impediment or interruption if selection for favored alleles also induces genomic change that results in negative fitness consequences at other loci (Schiffers et al. 2013; Edelman and Mallet 2021; Payne et al. 2024).

Specifically, one evolutionary process that can impede adaptive introgression is the loss of fitness that can occur when epistatically coevolved gene complexes are broken up by recombination, such as in Bateson-Dobzhansky-Muller incompatibilities (BDMIs; Bateson 1909; Dobzhansky 1936; Muller 1942; Orr 1995). During hybridization, coevolved gene complexes from divergent populations are separated and mixed up by recombination, often resulting in novel allelic combinations and lower organismal fitness (Fishman and Willis 2001; Edmands and Timmerman 2003; Matute et al. 2010; Renaut and Bernatchez 2011). An example is found in the coevolution of the nuclear and mitochondrial genomes, which both experience selection to maintain favorable combinations of the nuclear DNA (nDNA)- and mtDNA-encoded gene products which make up multimeric protein complexes that function in mitochondrial metabolic processes (Lynch 1997; Rand et al. 2004; Wallace 2010; Burton and Barreto 2012; Osada and Akashi 2012; Havird et al. 2015; Sloan et al. 2018; Hill et al. 2019; Hill 2020; Bettinazzi et al. 2024; Princepe and de Aguiar 2024). Therefore, while hybridization may permit introgression of nuclear genes favored for one trait, such as higher thermal tolerance, traits associated with mitonuclear interactions may suffer as a result of BDMIs if there is genetic linkage between causative loci for the two phenotypes. Conversely, mitonuclear coadaptations may reduce the degree of introgression of thermal tolerance alleles in nuclear genes linked to loci involved in mitochondrial function.

In this study, we used experimental evolution in reciprocal interpopulation hybrids of the copepod *Tigriopus californicus* to test how adaptive introgression of thermal tolerance alleles is impacted by variation in the degree of mitonuclear incompatibility. Populations of *T. californicus* are geographically isolated along the west coast of North America and show wide variation in high temperature tolerance consistent with local adaptation by latitude, with southern populations displaying increased tolerance relative to northern populations (Kelly et al. 2012; Leong et al. 2017; Pereira et al. 2017). Fitness breakdown is prominent among hybrids of *T. californicus*, with strong evidence that outbreeding depression and BDMIs are driven by mitonuclear incompatibility between populations (Edmands 1999; Willett 2006; Ellison and Burton 2008; Lima et al. 2019; Healy and Burton 2020). These factors suggest that *T. californicus* populations have experienced strong selection both for thermal tolerance (Pereira et al. 2014, 2017) and mitonuclear coevolution (Willett and Ladner 2009; Barreto et al. 2018; Pereira et al. 2021), and that adaptive introgression between populations under thermal stress could be negatively or positively impacted by fitness outcomes associated with highly coevolved mitonuclear gene complexes. A recent study by Griffiths et al. (2021) observed that in hybrids of *T. californicus*, alleles from a heat-tolerant southern population increased in frequency in response to thermal selection despite an mtDNA background from a heat-sensitive northern population. Their results hence provide evidence that adaptation to thermal stress can overcome potential fitness losses associated with mitonuclear incompatibility, though we argue that a clearer understanding of this interaction requires quantifying the phenotypic and genomic response in both directions of the hybrid cross to identify signatures of thermal selection and the impact that mitonuclear incompatibility has on them.

Here we performed selection for thermal tolerance in replicated populations of hybrids between copepods from San Diego, California (SD) and Strawberry Hill, Oregon (SH). These populations show marked differences in survivorship at high temperatures (Kayhani and Barreto 2023), with SD having higher tolerance than SH. They also exhibit extreme mtDNA sequence divergence (∼20.9%), which has resulted in recombinant hybrids that show phenotypic variation consistent with strong mitonuclear coevolution (Han and Barreto 2021). Han and Barreto (2021) showed that recombinant hybrids with low fitness in metabolic and developmental traits consistently have “mismatched” genomes (eg, mtDNA from SH and high nuclear allele frequencies from SD), congruent with findings in other crosses that mitonuclear incompatibilities play a major role in hybrid breakdown (Healy and Burton 2020, 2023a). Recent population genetic studies using this species’ annotated genome have shown that loci involved in thermal adaptation and those involved in mitonuclear coevolution are scattered across the 12 nuclear chromosomes that make up the *T. californicus* genome, and are often on the same chromosome as each other (Barreto et al. 2018; Lima et al. 2019; Healy and Burton 2020; Griffiths et al. 2021; Han and Barreto 2021). Specific loci, however, have not been explicitly identified, and thus levels of linkage can only be presumed at broader genomic scales (ie large regions or whole chromosomes). Following ten generations of selection for thermal tolerance in reciprocal hybrid populations, we predict that nuclear SD alleles will increase in frequency (introgress) across the genome relative to SH alleles, given their naturally adapted backgrounds. However, we expect that hybrid populations harboring the SH mtDNA background will suffer reduced genome-wide introgression of warm-adapted SD alleles because of co-introgression of SD genes that will form incompatible mitonuclear combinations with the SH mtDNA components.

## Results

### Phenotypic variation associated with evolved thermal tolerance

Experimental evolution was performed in populations of hybrids established from controlled crosses of large, outbred stocks of SD and SH copepods, generating F_1_ offspring of the reciprocal SD♀xSH♂ and SH♀xSD♂. Six replicated lines of each cross containing 100 to 500 individuals were maintained in either a selection regime, in which high-temperature stress was applied once per generation, or a control-temperature regime that propagated a random set of individuals each generation without high-temperature exposure. The experiment was terminated after 10 generations of selection, resulting in 21 independent lines (5 SD♀xSH♂ control, 6 SD♀xSH♂ selected, 4 SH♀xSD♂ control, and 6 SH♀xSD♂ selected) after losing three lines to population crashes. All lines were then maintained at standard culture conditions for two generations (∼2 months) to allow population sizes to increase and be phenotyped.

**Figure 1:**
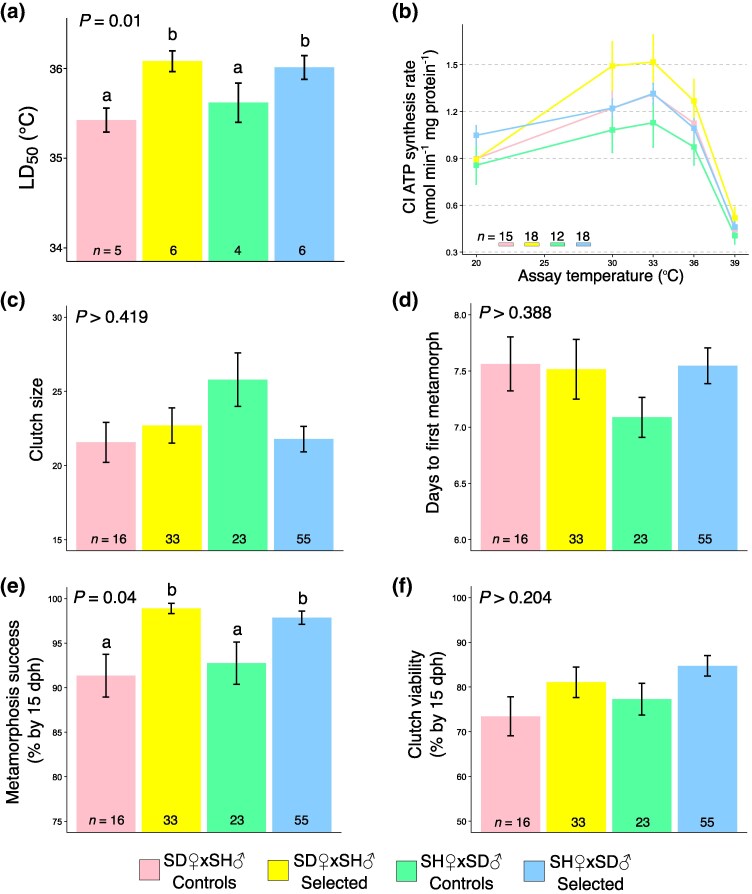
Phenotypic variation in heat-selected and control lines of reciprocal hybrids after ten generations of experimental evolution. a) Median lethal temperature (LD_50_) following 1-h stress in a temperature gradient. Plotted are mean ± SEM across experimental lines in each group. A total of 60 adult male copepods were used from each line. b) Complex I-driven ATP synthesis rates across temperatures quantified in vitro from isolated mitochondria. Plotted are mean ± SEM per group estimated across three technical replicates per line. Changes in rate between adjacent stress temperatures were assessed by Welch's two-sided *t*-tests and by Cohen's *d* effect size tests within each experimental group (see Table S2 for complete statistical results). c) Count of hatched nauplii in a first clutch. d) Number of days post hatch (dph) to first metamorphosed juvenile. e) Percentage of metamorphosis success per clutch at 15 dph. f) Percent of individuals per clutch alive at 15 dph. Plotted in (c to f) are mean ± SEM estimated from technical replicates from each line. Differences among experimental groups were tested with two-way ANOVAs (a), and linear mixed-effects models (c to f). *P*-values are based on each respective full model, and lower-case letters in (a and e) are from Tukey post hoc tests; different letters denote significance differences.

To capture change in thermal tolerance during selection, we estimated median lethal temperature (LD_50_) at the final generation F_12_. Experimental group was found to be a significant factor contributing to differences in LD_50_ (*F* = 4.8, *P* = 0.01), with Tukey post hoc and Cohen's *d* effect size tests suggesting that the selected lines of both crosses showed significantly higher LD_50_ temperatures than the control lines of both crosses (Fig. 1a; Table S1). These tests also showed that the crosses did not differ in the LD_50_ of their selected lines (*P* = 0.868, *d* = 0.35) or control lines (*P* = 0.909, *d* = 0.19).

We also tested whether the adaptive increase in thermal tolerance seen in selected hybrid lines was associated with a change in stability of mitochondrial performance during temperature ramping, as robustness of ATP synthesis to changes in temperature may indicate a form of thermal adaptation (Kayhani and Barreto 2023). We quantified the effect of temperature on Complex I (CI)-driven ATP synthesis rate in isolated mitochondria from F_12_ copepods (Fig. 1b). Within each experimental group, two-way ANOVA tests indicated that temperature had a significant effect on rate of ATP synthesis (SD♀xSH♂ selected: *F* = 9.828, *P* = 1.39 × 10^−6^; SH♀xSD♂ selected: *F* = 34.38, *P* < 10^−16^; SD♀xSH♂ controls: *F* = 12.86, *P* = 6.66 × 10^−8^; SH♀xSD♂ controls: *F* = 5.568, *P* = 7.76 × 10^−4^), and patterns of Cohen's *d* effect size of mean ATP synthesis rate across stress temperatures suggest that the two crosses differ in how this phenotype responded to selection (Table S2). In the SD♀xSH♂ cross, ATP synthesis rates changed less across temperatures in selected lines compared with their control counterparts, with the effect size of change in rate being larger in control replicates at the higher end of temperature changes (Table S2). The opposite pattern was observed in SH♀xSD♂, in which control lines appear to display less dramatic changes in ATP synthesis rates over increasing temperatures, especially at the high end of stress temperatures, suggestive of higher stability of synthesis rate (Table S2).

Heat selection experiments in a different *T. californicus* hybrid cross found evidence for a fitness tradeoff between tolerance and fecundity after five generations of selection (Kelly et al. 2016). As a consequence of potentially increased mitonuclear incompatibility, we also expected heat-selected lines of SH♀xSD♂ to show the lowest fitness levels in some of these phenotypes because of the predicted increase in SD nuclear alleles genome-wide. We quantified fecundity and three other life-history traits commonly assayed in this system and used linear mixed-effects models to test for differences among experimental groups. Because these assays were performed after sampling for genome sequencing, the number of individuals available was greatly reduced and variable among experimental lines. Each phenotype was measured in 1 to 12 technical replicates per line (median = 4). In contrast to Kelly et al (2016), we did not detect significant differences in fecundity (*P* > 0.419), developmental rate (*P* > 0.388), or clutch viability (*P* > 0.204) among experimental groups (Fig. 1c, d, and f). Metamorphosis success, however, was found to be significantly higher in selected lines compared with control lines (*P* < 0.04). Replicates from selected lines consistently approached 100% success in metamorphosis by day 15 post hatch, while control lines averaged 91% to 92% (Fig. 1e).

### Successful introgression of warm-adapted alleles despite the risk of increased mitonuclear incompatibility

We used a Pool-seq approach to quantify patterns of allele frequency changes at single nucleotide polymorphism (SNP) loci. We obtained a total of 2.81 billion paired-reads across the 21 experimental lines at F_12_, which resulted in high and similar depth of coverage across groups after read mapping (mean ∼153×; Table S3). Estimates of paternal leakage of mtDNA in the hybrid Pool-seq samples, if present, were extremely low (mean: 0.22%; range: 0.018% to 0.853%; Table S4) and cannot be distinguished from potential sequencing error rates (Stoler and Nekrutenko 2021).

**Figure 2:**
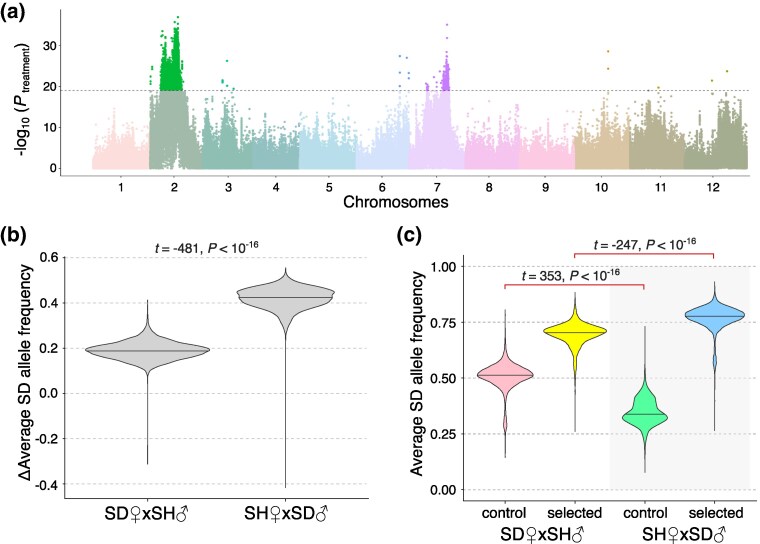
Genomic signal of high temperature selection regime. Shown are results quantifying treatment effect (heat-selected vs. control groups) after ten generations of experimental evolution. a) Manhattan plot of statistical tests for effect of selection treatment at each SNP locus (*N* = 2,425,402). The dashed line marks the threshold of the 1% most significant SNPs (*P* = 1.01 × 10^−19^), with brighter colors indicating SNPs above that threshold (*N* = 24,255 SNPs). b) Violin distributions of the change (Δ) between control and selected lines of each cross in average SD allele frequency, with black lines indicating the median of those values. Allele frequency averages were estimated within each hybrid cross and across replicate lines in each selection regime for each SNP locus detected above the significance threshold. c) Violin distributions of average SD allele frequencies in genomic regions under thermal selection across replicate lines within each experimental group, with black lines indicating the medians. These were the values used to calculate Δ in (b). Statistics reported are from paired *t*-tests.

A total of 2,425,402 SNP loci were retained in common across all samples. Allele frequencies across experimental groups were modeled with a generalized linear model with a binomial distribution (binomial GLM), incorporating a likelihood ratio test (Wiberg et al. 2017; Materials and Methods) to estimate the effect of selection regime (heat-selected or control; Fig. 2) on genome-wide allele frequency changes. Using a stringent genome-wide statistical threshold to identify the 1% of SNPs (24,255 SNPs) with the most significant changes in allele frequencies, we detected a significant effect of the thermal selection regime in seven of 12 chromosomes (hereafter termed “regions under thermal selection”). The vast majority of SNPs with significant allele frequency changes in response to thermal selection, however, were concentrated on Chromosomes 2 and 7 (>99.9%) (Fig. 2a). We examined the pattern of allele frequencies within these genomic regions in each experimental group to determine whether there was bias toward the SD parental allele. Selected lines of both crosses showed substantial increases in the average frequency of the SD allele relative to their respective controls. Notably, the magnitudes of their responses differed significantly (*P* < 2.2 × 10^−16^, paired *t*-test), with the SH♀xSD♂ cross showing a larger change in SD allele frequency (mean Δ = 0.42) between selected and control lines than the SD♀xSH♂ cross (mean Δ = 0.19) (Fig. 2b). To examine the nature of this difference, we compared the distributions of SD allele frequencies between the control lines and between the selected lines. SD allele frequency in SD♀xSH♂ controls increased slightly above 0.5 (to 0.51) after 12 generations. SH♀xSD♂ controls, however, showed frequencies of the SD allele that were, on average, 0.16 lower than those of the reciprocal (*P* < 2.2 × 10^−16^, paired *t*-test; Fig. 2c), indicating that these lines increased in the frequency of their maternal SH allele, rather than the SD allele. In contrast, selected lines of SH♀xSD♂ had significantly higher SD allele frequencies (by 0.07) than SD♀xSH♂ selected lines (*P* < 2.2 × 10^−16^, paired *t*-test; Fig. 2c). Therefore, the higher change in allele frequency (mean Δ) between SH♀xSD♂ treatments was a result of both an increase in SD alleles during heat selection and increase in SH alleles in the absence of heat selection (Fig. 2b). Interestingly, the distribution of SNP frequencies in the non-significant loci (the 99% below the statistical threshold) shows a general bias toward the maternal background, consistent with selection to maintain mitonuclear match (Fig. S1).

Since genome-wide patterns across the regions under thermal selection are heavily influenced by just two chromosomes, we examined allele frequencies on all seven chromosomes individually. The general pattern of increased SD alleles in selected lines is recapitulated independently on Chromosomes 2, 3, 7, 11, and 12 (Fig. S2), as well as the pattern of lower SD allele frequency (higher SH frequency) in SH♀xSD♂ controls relative to the reciprocal controls (Figs. S2 and S3). This suggests that in most chromosomes that showed a significant effect of temperature selection, the predominant pattern indicates that the SD allele increased in frequency in response to thermal selection and that the maternal allele increased in frequency in the controls of both crosses. In Chromosomes 6 and 10, the inverse pattern is seen, where both selected lines show a decrease in SD allele frequency (an increase in SH frequency) relative to what happened in the control regime (Figs. S2 and S3). Although this latter pattern is found in regions with a relatively weak signal, it is indicative of the presence of some loci in which the SH allele is selected for over the SD allele in adaptation to high temperatures.

Even within single chromosomes, averaging mean allele frequencies across all loci under selection has the potential to disregard genetic linkage and mask differences in the direction of allele frequency changes between linkage blocks. Therefore, we further examined how the patterns of allele frequency change were distributed across blocks within Chromosomes 2 and 7. We identified the single most significant SNP within each of 16 non-overlapping 500-kb windows on Chromosome 2 (Fig. S4a) and within 17 non-overlapping 250-kb windows on Chromosome 7 (Fig. S4c). Allele frequencies based on these SNPs reiterated the chromosome- and genome-wide pattern (Fig. S4b and d), strengthening the evidence for SD allele introgression across multiple regions of the chromosome in both crosses when under high-temperature selection.

We chose to use a 1% genome-wide statistical threshold to prioritize regions of highest impact. This threshold is more stringent than the widely used Bonferroni correction, which would have retained over eight times as many SNP loci (209,864, Fig. S5). To assess whether the patterns of allele frequency are affected by the choice of threshold, we examined the SNP frequencies across chromosomes using the Bonferroni threshold. We found strong concordance in patterns revealed by the two thresholds. The additional ∼185,000 loci permitted by the Bonferroni approach showed the same pattern across individual chromosomes as the 1% threshold (Fig. S6), suggesting that our stricter approach recapitulates the response appropriately while introducing fewer low-impact genomic regions.

### Functional annotation of regions under thermal selection

Intersection of the 24,255 SNPs under thermal selection with the reference genome identified 15,059 instances where a SNP overlapped the coordinates of at least one gene and 9,954 SNPs that are intergenic. Among the 15,059 total genic SNP matches, we identified 598 unique genes across the regions under selection (Table S5), with most (536) located on Chromosome 2. With such a high concentration of genes within few broad peaks, it is likely that most genes are neutral with respect to the selection response and are linked to relatively few loci under selection within these regions. We further examined the genic SNPs to assess whether they were randomly distributed between exons and introns. Although more intronic than exonic SNPs were detected overall on Chromosome 2 (7,816 intronic vs. 6,606 exonic), the number of exonic SNPs is significantly higher than expected by chance when considering the total landscape of exons and introns (Fisher's exact test: odds ratio [OR]: 1.10, 95% confidence interval [CI]: 1.06 to 2.14, *P* = 5.31 × 10^−9^). This trend was even more robust when only the most significant single SNP per gene was considered (OR: 2.26, 95% CI: 1.86 to 2.71, *P* = 1.21 × 10^−20^), suggesting that the SNPs that experienced the most significant changes in allele frequency are more likely than by chance to occur in exons.

Additionally, we examined the 598 genes detected above for possible overrepresentation of cellular functions. Among the top 50 most enriched terms based on gene ontology (GO) biological processes, several have broad roles in cellular stress, including DNA damage response (GO:0006974), protein ubiquitination (GO:0070534; 0016567; 0051865; 0035871), stress-activated protein kinase cascade (GO:0007254), and regulation of response to external stimulus (GO:0032101). Several of these terms were associated with stress response of the endoplasmic reticulum (ER) specifically (eg “regulation of IRE1-mediated unfolded protein response,” and “ubiquitin-dependent ERAD pathway”) (Table S6). We also manually checked for the presence of genes encoding heat-shock proteins and found two *DnaJ* (*Hsp40*), one small *Hsp* (*Hsp26*), and a major chaperone of the ER stress response (*HSPA5*). As a group, however, *Hsp* genes were not overrepresented among the 598 genes in our list (*P* = 0.54; Fisher's exact test). Other known stress-response genes in this list include carbonic anhydrase (osmoregulation, acid-base balance), thioredoxin and glutathione *S*-transferase (antioxidant response), and calreticulin (protein folding in the ER) (Table S5).

Finally, to assess whether adaptation to heat stress altered allele frequencies at genes involved in mitonuclear interactions, we cross-listed the 598 genes above against a set of nuclear-encoded proteins known to interact directly with mtDNA-encoded products (Burton and Barreto 2012; Barreto et al. 2018). Interestingly, we detected 6 mitochondrial ribosomal proteins (mt-RPs) from a total of 67 in the genome, though this was not more than expected by chance (*P* = 0.17; Fisher's exact test). This is of note because mt-RPs have been suggested as important players in the maintenance of mitonuclear matching in multiple organisms (Barreto and Burton 2013; Li et al. 2024; Nikelski and Weir 2025), and we hence expected these to increase in SD frequency only in the cross with SD mtDNA, not in both.

### Localized but strong mitonuclear coevolution alongside thermal selection

**Figure 3:**
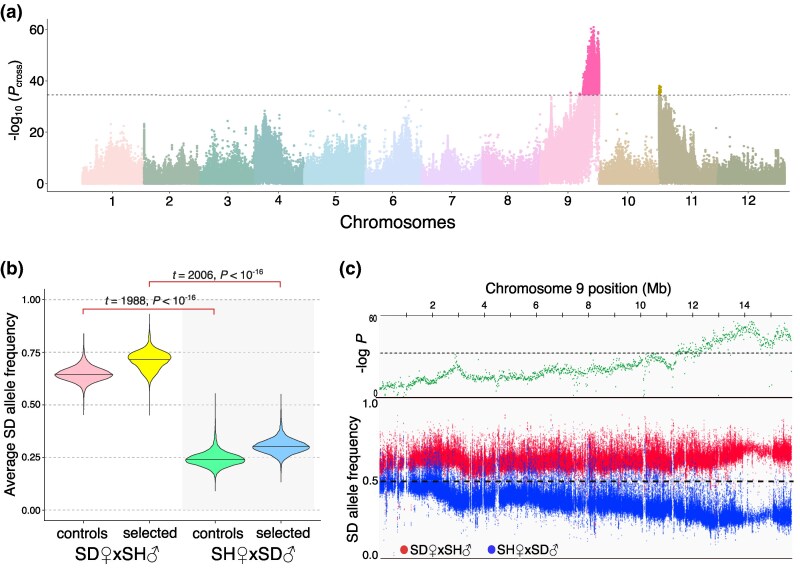
Genomic signal of cross direction. Shown are results quantifying the effect of cross direction (SH♀xSD♂ vs. SD♀xSH♂) after ten generations of experimental evolution. a) Manhattan plot of statistical tests for cross effect in each SNP locus (*N* = 2,425,402). The dashed line marks the threshold of the 1% most significant SNPs (*P* = 2.61 × 10^−35^), with brighter colors indicating SNPs above that threshold (*N* = 24,255 SNPs). b) Violin distributions of SD allele frequencies in genomic regions with significant change due to cross direction, averaged across replicate lines within each group, with black lines indicating the medians. Statistics reported are from a paired *t*-test. c) Distribution of allele frequencies (*bottom* panel) and model *P*-value (*top* panel) along Chromosome 9. Data were visualized in Integrated Genomics Viewer 2.19.4, with *P*-value data plotted as maximum values in windows (using the “Windowing Function” in IGV). Dashed horizontal line marks the statistical threshold as in (a).

Following our main analysis, we extracted the effect of cross direction (SD♀xSH♂ or SH♀xSD♂; Fig. 3) from the statistical model to identify regions of the genome responding to selection for mitonuclear compatibility alongside thermal selection. Recent work mapping the impact of mtDNA background on the nuclear genome of *T. californicus* found evidence of widespread mitonuclear coadaptation in early-generation F_2_ hybrids. These studies reported that fitness variation was associated with strong mitonuclear matching in two to five chromosomes across four different interpopulation crosses (Lima et al. 2019; Healy and Burton 2020; Han and Barreto 2021). Given the clear pattern of SD nuclear allele introgression in heat-selected hybrids with SH mtDNA (SH♀xSD♂) observed above, we asked what genomic regions may still be targets of mitonuclear coadaptation. Examining the cross effect in the full model on F_12_ allele frequencies, we applied the same genome-wide threshold as above to identify the 1% of SNPs (24,255 SNPs) with the most significant allele frequency changes based on cross direction (but not thermal selection regime). These SNPs may then represent regions of the genome where mitonuclear incompatibilities were alleviated in each experimental group by selection for coadapted genes regardless of treatment. All of the SNP loci above this threshold were located on Chromosomes 9 (24,235 SNPs) and 11 (20 SNPs) (Fig. 3a). On Chromosome 9, the SNPs are concentrated on a relatively narrow region at one end of the chromosome (positions 10,489,460 to 15,792,794). When allele frequencies were examined in these loci, we observed a strong pattern of mitonuclear matching; both selected and control lines of each cross showed increased nuclear allele bias toward their respective maternal populations (Fig. 3b). This trend was clear even outside of the region of statistical significance along the entire length of Chromosome 9 (Figs. 3c and S3). Direct comparison of allele frequencies between crosses revealed that they diverged by ∼0.40 in the frequency of SD alleles in control (paired *t* = 1,988, *P* < 2.2 × 10^−16^) and selected lines (paired *t* = 2,006, *P* < 2.2 × 10^−16^) (Fig. 3b). The signal of strong mitonuclear matching was robust after testing for linkage effects using 500-kb non-overlapping windows for Chromosome 9 (Fig. S7b) and when Chromosome 11 is inspected on its own (Fig. S7c and d).

We identified 10,910 genic SNP matches in these regions, corresponding to 306 unique genes under selection due to cross direction (Table S7). Here we were particularly interested in assessing the presence of nuclear-encoded mitochondrially targeted proteins (MTPs), which comprise components of numerous biochemical pathways within the organelle. Among the 306 genes within these regions, we identified 7 MTPs out of a possible 600 annotated in the *T. californicus* genome (Barreto et al. 2018), though this was not more than expected by chance (*P* = 0.99; Fisher's exact test). Among MTPs, only ∼25% of proteins perform their functions via direct interactions with mtDNA or mtDNA-encoded elements. These functions include mtDNA transcription, ribosome formation, translation, and OXPHOS (Burton and Barreto 2012). This latter set of mtDNA-interacting proteins have been shown to evolve faster than the non-interacting subset, presumably as a result of coadaptation to rapidly diverging mtDNA (Barreto et al. 2018; Weaver et al. 2022; Biot-Pelletier et al. 2023). The *T. californicus* nuclear genome harbors 146 putatively mtDNA-interacting protein genes that are distributed across the 12 chromosomes (Barreto et al. 2018). Of note, one of the seven MTPs (and the only mtDNA-interacting protein gene) within the region examined here was *COX6A1*, an nDNA-encoded protein subunit of Complex IV of the OXPHOS pathway.

Finally, when assessed via GO annotation, the 306 genes seem to have a disproportionate number of roles in cellular and organismal development. For example, among the top 50 biological process terms significantly overrepresented, 29 are associated with “morphogenesis,” “development,” “cell fate,” “differentiation,” and “organ formation” (Table S8). No *Hsp* genes were found in this list.

## Discussion

Adaptive introgression of thermal tolerance-associated alleles offers the potential for genetic rescue of less tolerant populations through hybridization (Bell 2013; Hedrick 2013), but the success of introgression can be influenced by selection against those alleles if they are unfavorable for other aspects of the environment or linked to genes under opposing selection (Schiffers et al. 2013; Sachdeva and Barton 2018). Results from the current study suggest that between the SD and SH populations of *T. californicus*, which have evolved under divergent thermal regimes in nature, selection for thermal tolerance and selection for compatible mitonuclear combinations are both strong forces shaping the nuclear genome. In this study, adaptation to high temperature stress was able to proceed via introgression of warm-adapted alleles despite the risk of fitness losses due to mitonuclear incompatibility on an increasingly hybridized genome, at least in the short term of approximately ten generations of evolution. Replicated lines of both SD♀xSH♂ and SH♀xSD♂ responded to selection by evolving increased thermal tolerance relative to their respective control lines, with no significant differences in the magnitude of response between crosses. Allele frequencies of loci under selection in the current study corroborate that improved thermal tolerance in the selected lines is largely associated with increased proportions of SD alleles. These findings are concordant with phenotypic and genetic outcomes of an earlier experiment using a different *T. californicus* hybrid cross (Griffiths et al. 2021), which showed widespread introgression of SD alleles in a hybrid with a maternal background from a cooler climate. By examining both crosses reciprocally, our study provides added power to understand the risk of potential fitness tradeoffs of adaptive introgression and identify genomic regions under selection that may play a disproportionate role in resolving mitonuclear incompatibility in a short-term period of adaptive evolution.

Comparison of genomic change between the reciprocal crosses further revealed that, contrary to our prediction, SD allelic proportion was not lower in selected lines of SH♀xSD♂ relative to the reciprocal cross. This suggests that genome-wide introgression of heat-adapted SD alleles was at least equally successful in the SH♀xSD♂ selected lines, despite this cross direction having increased risk of mitonuclear incompatibilities due to a greater proportion of SD nuclear alleles introduced onto a maternally inherited SH mtDNA background. The potential for mitonuclear incompatibilities was supported by the pattern shown in the control lines of SH♀xSD♂, which accumulated more maternal SH alleles beyond the initial frequency of 0.5 in the same loci responding to heat selection in the selected group. This demonstrates that mitonuclear coadaptation is strong in this cross direction, making the marked increase in SD alleles during thermal adaptation particularly remarkable. Using reciprocal crosses from the same populations, Han and Barreto (2021) also found evidence for strong mitonuclear coadaptation, showing that high-fitness but not low-fitness F_2_ hybrids have nuclear and mitochondrial genomes matched for the same population of origin. Their genomic data, however, showed mitonuclear effects on many chromosomes, especially Chromosomes 1, 4, and 9, whereas our study captured patterns only on Chromosomes 9 and 11. Many differences in experimental design and analyses (different generations, selection regimes, statistical approach, replication) likely explain much of this contrast between studies, but it is also possible that our study detected only genomic regions that were more free to develop mitonuclear matching during the temperature selection regime.

If both thermal tolerance and mitonuclear fitness are highly polygenic, it is unlikely that recombination through ten generations is sufficient to segregate causative loci between these traits. We hence argue that the pattern we observed indicates that thermal tolerance or mitonuclear fitness may be sufficiently controlled by a small number of genes, allowing for the breakup of linkage to occur relatively early in hybridization in this system. The genomic regions identified here were broad and encompass many genes, likely reflecting hitchhiking around a limited number of causal loci rather than widespread independent targets of selection. Indeed, previous experimental work monitoring intrinsic fitness in *T. californicus* hybrids found that hybrids lines that experience early hybrid breakdown in fitness could often recover within nine generations, and that these changes were associated with increased frequencies of maternal alleles in reciprocal crosses, hence indicating sufficiently rapid breakup of linkage (Pereira et al. 2021). In contrast, Griffiths et al. (2021) estimated recombination rates to be relatively low in *T. californicus* during experimental heat selection and argue that any conflicting mitonuclear selection is simply being overwhelmed by extrinsic selection in heat-selected lines, despite linkage being maintained. Further supporting this, losses in hybrid fitness due to mitonuclear incompatibility in *T. californicus* have been shown to be ameliorated when hybrids are exposed to chronic heat stress (Willett 2012), suggesting that the benefits of introgression outweigh the costs when thermal stress is high. While the present study is unable to directly compare the strengths or selection coefficients of these two forces, our results contribute to the broader observation that improved thermal fitness as a result of genome-wide introgressive hybridization can occur rapidly despite the severe costs of negative epistatic interactions or outbreeding depression that commonly occur in early hybrid generations in this system. More importantly, our efforts to partition the effects of heat selection from mitonuclear coadaptation in reciprocal hybrids inform us about the genomic architecture of these forces and how a possible fitness tradeoff may be resolved via separation of few high-impact loci.

Genes within regions that responded to heat selection comprise several groups with known roles in stress response. We identified genes with roles in protein ubiquitination and proteolysis, as well as four *Hsp* genes involved in molecular chaperoning, all of which are crucial mechanisms of homeostasis during cellular stress (Lindquist 1986; Wilkinson 1995; Feder and Hofmann 1999; Pickart 1999). In all of the genes implicated in the functions above, there was strong SD allele bias during selection for increased heat tolerance in selected lines of both crosses in our study, which is consistent with the expectations that alleles of SD origin provide a substantial increase in fitness during regimes of frequent high temperatures (Leong et al. 2017; Pereira et al. 2017; Griffiths et al. 2021; Healy and Burton 2023b), and that this introgression occurred in important stress genes, among others. The observed introgression of SD alleles is less clear, however, in genes involved in mitonuclear interactions. Despite resulting in mitonuclear mismatch in SH♀xSD♂ selected lines, six mt-RPs, all on Chromosome 2, evolved higher SD allele frequencies in response to heat selection. Chromosome 2 also carries six additional mt-RPs that showed the same pattern but did not cross our statistical threshold. In control lines, regions with mt-RPs instead showed an increase in the allele of their respective maternal origin (Fig. 2), suggesting they experienced selection for favorable mitonuclear matching. Mt-RPs and rRNAs form mitoribosomes that function exclusively to translate mtDNA-encoded proteins involved in OXPHOS. The bias we observed toward SD alleles may reflect that thermal stability in mitochondrial ribosomes, enhanced by SD alleles, is adaptive in thermally stressful environments to optimize mitochondrial translation regardless of mtDNA background. Alternatively, these mt-RP genes may simply experience relatively relaxed mitonuclear selection compared with other loci (such as OXPHOS subunits) and thus increases in SD allelic proportion at these loci may simply be due to proximity or linkage to other genes experiencing stronger selection for thermal tolerance.

Our results contribute to a relatively recent body of work examining the role of mitochondrial evolution in the thermal tolerance of ectotherms. Much of this work has focused on the mitochondrial climatic adaptation hypothesis, which is based on evidence of thermal adaptation in mitochondrial genomes and contributions of mitochondrial haplotypes on the upper thermal limits of organisms (Chung and Schulte 2020). In *Drosophila*, mitochondrial haplotype frequency has been demonstrated to respond to temperature selection (Lajbner et al. 2018), and sequence variation in mitochondrial haplotypes contributes to organismal thermal tolerance consistent with local adaptation by latitude (Camus et al. 2017). When considering mitonuclear coadaptation, mismatch between locally adapted mtDNA haplotypes and nuclear genomes in hybridized flies resulted in increased respiratory rates and oxygen consumption, but at the cost of reduced fecundity and survival (Bettinazzi et al. 2024), suggesting that divergence between populations as a result of local adaptation to temperature poses serious fitness risks for hybrids at mitonuclear loci. Among specific loci shown to impact thermal tolerance, genes encoding subunits of the cytochrome *c* oxidase complex (COX) have been implicated as potential sites in *Drosophila* where nonsynonymous differences between mtDNA haplotypes are correlated with tradeoffs between enzyme activity and cold tolerance (Ballard et al. 2007). Similarly, studies in *Saccharomyces* yeasts have also demonstrated that mitochondrial haplotype, and specifically divergence in COX proteins, directly contributes to differences in thermal tolerance between reciprocal hybrids (Baker et al. 2019; Li et al. 2019). Recent work in non-model arthropods also provides evidence that mtDNA and *COX* genes can evolve in response to thermal selection (Li et al. 2018; Guerin et al. 2024) and that mitochondrial physiology during thermal stress is impacted by local adaptation (Havird et al. 2020; Menail et al. 2022). In contrast, our results suggest that in *T. californicus*, *COX* genes play a larger role in mitonuclear coevolution than they do in local adaptation to temperature, as we only identified one COX member (*COX6A1*) within regions responding to selection, but due to mitochondrial background, not thermal selection.

Natural populations of *T. californicus* show local adaptation in thermal sensitivity of mitochondrial performance (Harada et al. 2019) that is correlated with organism upper thermal limits (Healy and Burton 2023b). Our recent experimental work, however, identified that mtDNA background alone does not underpin the differences in survival or Complex I (CI)-driven ATP synthesis between the SD and SH populations during thermal stress (Kayhani and Barreto 2023). Mitochondria from pure SD individuals display less dramatic changes in the rate of ATP synthesis over increasing temperatures than pure SH or F_1_ hybrid individuals of both cross directions, indicating that any local adaptation in the thermal stability of ATP synthesis is likely due to increased dosage of SD nDNA-encoded proteins functioning within the mitochondria instead of SD mtDNA alone (Kayhani and Barreto 2023). In the present study, when we compare the two groups under thermal selection, we find that SH♀xSD♂ selected lines show lower stability of CI-driven ATP synthesis rate over increasing temperatures than either the reciprocal (SD♀xSH♂ selected) or its counterpart control group (SH♀xSD♂ controls) (Table S2). This could be a byproduct of broad introgression of SD alleles resulting in some incompatibility between SD nDNA-encoded products and SH mtDNA-encoded products acting within the mitochondria (Ellison and Burton 2006; Ellison and Burton 2008; Healy and Burton 2020; Han and Barreto 2021). While we did not find any genes that encode subunits of OXPHOS complexes (such as *COX* genes) to be responding to thermal selection, we did identify six genes that encode mt-RPs, which are responsible for the translation and expression of OXPHOS subunits (Ott and Herrmann 2010). Mitochondrial translation has been proposed as a function impacted by mitonuclear incompatibility (Hill 2017; Hill et al. 2019), and these genes evolve rapidly in *T. californicus* (Barreto and Burton 2013). Reduced stability of ATP synthesis rates in SH♀xSD♂ selected lines could potentially be the result of reduced efficiency in translation of OXPHOS gene transcripts by ribosomal complexes that harbor mixed mitonuclear components. Nevertheless, the level of variation in stability of CI-driven ATP synthesis rate we observed does not appear to predict differences in survivorship.

We found evidence that the nDNA-encoded *COX6A* can respond to selection to maintain match with the mtDNA, which may point to a key role for *COX* genes in resolving potential mitonuclear fitness tradeoffs during adaptive introgression. The high degree of mitonuclear matching localized to Chromosome 9 (Fig. 3) suggests that a few loci of strong effect may be sufficient to maintain a balance between thermal tolerance and mitonuclear function, again assuming that most genes in this broad region are hitchhiking and are not direct targets of selection. We propose that among genes predicted to be involved in crucial mitonuclear interactions, *COX6A1* on Chromosome 9 may impart a disproportionate impact on mitonuclear functions. This gene contained SNPs with highly significant divergence in allele frequency, and it was the only one in this region that is predicted to rely on physical interactions with mtDNA-encoded proteins. *COX6A1* is one of six nuclear genes in *T. californicus* encoding subunits of COX, the final enzyme of the mitochondrial respiratory chain (Barreto et al. 2018). These protein subunits form extensive interactions with three large subunits encoded by the mtDNA (COX1–COX3). Besides evidence of rapid molecular evolution in COX subunits (Osada and Akashi 2012; Barreto et al. 2018), impairment of COX enzyme activity due to mitonuclear incompatibilities has been experimentally demonstrated in multiple systems, including rodents (Yamaoka et al. 2000), primates (Osheroff et al. 1983; Barrientos et al. 2000), and copepods (Rawson and Burton 2002; Harrison and Burton 2006). In *T. californicus*, mitonuclear mismatches in COX subunits also affect fitness in fecundity and viability, in at least some interpopulation crosses (Edmands and Burton 1999; Willett and Burton 2001). We speculate that mitonuclear coadaptation in the COX enzyme may be particularly strong in the SD and SH populations, and that matching of *COX6A1* to the maternal background may sufficiently rescue mitonuclear function such that heat selection can work efficiently in other regions of the genome harboring more stress-response functions, such as Chromosome 2. *COX6A1* does not appear to be linked to any obvious stress-response genes, permitting recombination to separate the two functions in relatively few generations. Functional and fitness experiments on the disproportionate impact of *COX6A1* in SDxSH hybrids are warranted and feasible.

Reduced metamorphosis success, clutch size, viability, and developmental rate have long been used as measures of fitness in *T. californicus* (Burton 1990; Edmands et al. 2009), and are frequently associated with mitonuclear incompatibility in hybrids (Ellison and Burton 2008; Healy and Burton 2023a). In the present study, we expected heat-selected lines to show a decrease in some of these life-history traits relative to control lines because of fitness tradeoffs, as observed for fecundity by Kelly et al. (2016) with hybrids selected for heat tolerance for five generations. We found that selected lines of both crosses actually showed robustness in metamorphosis success, suggesting that selection in both crosses may have led to a positive pleiotropic effect on general fitness. Among the other phenotypes, the lack of pattern and wide variance in data suggest that these traits are not only highly polygenic, but also strongly influenced by the many aspects of the environment besides acute temperature (eg density and nutrition). It is also possible that, if heat-selected alleles are few and largely unlinked from the other phenotypes, ten generations of selection may have alleviated moderate fitness tradeoffs present in early generations. Finally, SH♀xSD♂ selected lines were predicted to be the most susceptible to mitonuclear incompatibility as a result of genome-wide introgression of SD alleles. However, this group did not show fitness losses across life-history phenotypes compared with other experimental groups and showed improved metamorphosis success compared with its control counterpart. This suggests that loci under thermal selection may have segregated from those associated with these phenotypes via early recombination, though increased SD allele frequency in selected lines may be correlated with developmental success.

## Conclusion

The current study examined the capacity for adaptive introgression of thermal tolerance alleles between two highly divergent populations at the risk of inducing fitness tradeoffs from unfavorable mitonuclear allelic combinations. Our results demonstrate that, while both processes play a role in the evolution of hybridizing populations under thermal stress, selection for thermal tolerance, in this case, induced adaptive introgression despite the potential for fitness losses due putatively to increased mitonuclear incompatibilities in a hybridized genome. We note that cross-direction effects in reciprocal hybrid experiments can also arise from other maternally inherited cytoplasmic factors, thus we cannot rule out their relative contributions without targeted investigation. In regions of the genome responding to thermal selection, control lines of both crosses actually showed signatures of selection favoring their maternal parent (corresponding, eg, to maternally inherited mitochondria), while selected lines showed increased organismal tolerance and broadly induced introgression of SD alleles, regardless of cross. Loci evolving under thermal selection were identified across multiple chromosomes. However the majority were concentrated in Chromosome 2, suggesting genes in this region are potential sites where selection against mitonuclear incompatibilities in hybrids are outweighed by selection for thermal tolerance in stressful environments. Highly significant SNPs under heat selection were more likely to occur in protein coding sequences than regulatory regions, suggesting variation between populations is due to structural differences in genes. Variation in stability of CI-driven ATP synthesis between selected lines suggests that introgression resulting in mitonuclear mismatch could be impacting thermal stability of mitochondrial function, though this was not reflected in other measures of fitness, such as early-life viability or metamorphosis success. Adaptive introgression in the face of increasing temperature stress regimes thus may offer a form of genetic rescue for less tolerant populations through hybridization, even in cases where mtDNA divergence is high. This process can occur if fitness gained from warm-adapted alleles outweighs losses caused by mitonuclear incompatibility, or if those incompatibilities can be resolved relatively early through intrinsic selection acting on mitonuclear loci of highest impact. The genomic architecture required for maintenance of mitonuclear compatibility during introgression appears to be relatively simple in this case; in hybrids between SD and SH, OXPHOS *COX6A1* showed strong bias toward its maternal allele, which we suggest may have repaired mitonuclear function in hybrids such that adaptive introgression of heat-adapted SD alleles could occur in other regions of the genome without incurring major risks of mitonuclear incompatibility.

## Materials and methods

### Experimental evolution

All experimental populations were established using *T. californicus* stocks of the SD (San Diego, CA, USA, 32.45°, −117.25°) and SH (Strawberry Hill, OR, USA, 44.25°, −124.11°) populations that had been maintained in standard culture conditions (20 °C, 12 h:12 h light:dark) for at least two generations prior to use in experiments. Reciprocal crosses (SD♀xSH♂ and SH♀xSD♂) were formed by crossing 100 unmated females from each stock population with males from the other population, producing a large number of F_1_ hybrids that were used to initiate selection regimes. Six lines were established each with 110 female copepods in 400-ml beakers for the following treatments: SD♀xSH♂ heat-selected, SD♀xSH♂ controls, SH♀xSD♂ heat-selected, and SH♀xSD♂ controls. Experimental evolution for thermal tolerance in the 12 heat-selected lines was conducted through the F_10_ generation by exposing each line to a stress temperature for 1 h in a pre-heated water bath (starting at 35.7 °C for the F_1_ generation and increasing by 0.1 °C every subsequent generation). The 12 control lines were maintained in incubators under standard conditions and new generations were established by haphazardly subsampling mated females from each beaker to simulate the average amount of mortality seen in the heat-selected lines of that cross during that generation. In each replicate, generations were kept separate, with up to 250 individuals (median = 173) used to found each new generation. Following the F_10_ generation, all replicates were kept in standard conditions for two generations without selection to allow population sizes to increase so that genomic and phenotypic sampling could be performed. During the experiment one SD♀xSH♂ control and two SH♀xSD♂ control replicates experienced population crashes, resulting in 21 total replicates. Additional details can be found in the Materials and Methods.

### Phenotypic assays

The median lethal temperature (LD_50_) of each replicate line was measured during the F_4_, F_8_, and F_12_ generations by exposing ten individual male copepods to each of six stress temperatures ranging from 34.5 °C to 37 °C in 0.5 °C increments (*N* = 60 copepods total). Since LD_50_ was measured at intermediate generations, we used only males, and saved mated females, to minimize negative impacts on the production of subsequent generations. Thermal stability of ATP synthesis in the F_12_ generation of each line was assessed using a modified protocol from Kayhani and Barreto (2023) (Materials and Methods). Following the F_12_ generation, each line was tested for life history traits including fecundity (number of nauplii hatched from the first clutch), developmental rate (number of days to metamorphosis), viability (proportion alive at day 15 post-hatch), and metamorphosis success (proportion metamorphosed at day 15 post-hatch) (Materials and Methods).

### Determining genomic regions under selection

Genomic DNA from pools of ∼80 adult copepods was isolated from each replicate during the F_12_ generation, used to prepare whole-genome sequencing libraries, and sequenced as short-reads to a minimum read depth of ∼80×. Sequenced reads were mapped to a “hybrid” reference genome following an adaptation of the pipeline in Healy and Burton (2023a) (Materials and Methods) and SNP positions that are fixed between the SD and SH populations were retained for statistical analyses, resulting in 2.4 million SNP loci. SD and SH allele frequencies were then modeled using a binomial GLM incorporating a likelihood ratio test (Wiberg et al. 2017) to estimate the effect of treatment (selection or control) and cross (SD♀xSH or SH♀xSD♂) on those frequencies, after accounting for the interaction effect (treatment × cross). The 1% of SNPs with the smallest *P*-values for each effect were considered regions of the genome likely evolving due to each selective pressure. This statistical threshold is more conservative than genome-wide Bonferroni adjustments. Significant positions were then checked against the *T. californicus* SDv2.1 genome annotation (Barreto et al. 2018) to identify SNPs within protein-coding genes. Functional annotations of these genes were then mined to test for enrichment of cellular functions based on GO terminology with topGO (v2.54.0; Alexa and Rahnenfuhrer 2024). We also examined the presence of genes encoding proteins associated with heat-shock response and mitochondrial targeting (Materials and Methods).

Since paternal leakage of mtDNA has been reported in certain *T. californicus* hybrid crosses (Lee and Willett 2022), we examined whether this phenomenon may be occurring in our samples. For this, the mapping files of hybrid samples were briefly examined by counting number of reads mapping to each of the two mitotypes. These counts were compared with the mapping of reads from pure parental datasets from SD and SH (NCBI accessions: SD, SRR27277355 and SRR27277345; SH, SRR9019163) to estimate a background level of read mapping to the alternative mitotype. More details available in the Materials and Methods.

## Supplementary Material

msag158_Supplementary_Data

## Data Availability

Sequence data generated in this study are available from the NCBI Sequence Read Archive under BioProject accession PRJNA1054752.
