# Supplementary material for: Introgression of thermal tolerance alleles drives adaptation despite risk of mitonuclear conflict
Source: Mol Biol Evol. 2026 Jul 9;43(7):msag158. doi: 10.1093/molbev/msag158 (PMC13381279; doi:10.1093/molbev/msag158)
Supplement: msag158_Supplementary_Data [file msag158_supplementary_data.zip › Kayhanietal2025_Supplement_2ndRevision.pdf]

Supplementary Information for

INTROGRESSION OF THERMAL TOLERANCE ALLELES DRIVES ADAPTATION DESPITE RISK OF MITONUCLEAR CONFLICT

Kamron L. Kayhani[1]*, Jasmine R. Weaver[1,2], Molly K. Burke[1], Felipe S. Barreto[1]

[1]Department of Integrative Biology, Oregon State University, Corvallis, OR 97331, USA
[2]Current address: Department of Biological Sciences, University of New Brunswick-Saint John, Saint John, NB, Canada, E2L 4L5

*Corresponding author: kayhanik@oregonstate.edu; kamron.kayhani@gmail.com

In this file:
- Supplementary Materials and Methods
- Supplementary Tables S1-S4
- Supplementary Figures S1-S7.

Additional supplementary files:
- Supplementary Tables S5-S8 in one Excel file.

Supplementary Materials and Methods

Copepod culturing, hybrid crossing, and experimental evolution

All experimental populations were established using *Tigriopus californicus* stocks of the SD (San Diego, CA, 32.45°, -117.25°) and SH (Strawberry Hill, OR, 44.25°, -124.11°) populations that had been maintained for at least two generations prior to use in the creation of hybrid lines. All populations were maintained in 400-ml beakers containing 35 parts-per-thousand (ppt) salinity artificial sea water (ASW; Instant Ocean, Blacksburg, VA) on a diet of live *Isochrysis* and *Nannochloropsis*, and ground dried *Spirulina* wafers (Hikari Sales U.S.A, Hayward, CA) under common garden conditions in incubators at 20°C and with a 12 hr light:dark cycle (Thermo Fisher Scientific, Marietta, OH).

Reciprocal hybrid crosses (SD♀xSH♂ and SH♀xSD♂) were formed by crossing 100 unmated females from each stock population with males from the other population. Unmated females were obtained by removing them from mate-guarding pairs using a fine needle, introducing them to males of the reciprocal population, and allowing them to mate freely. Parent-generation females for each cross were kept in petri dishes (with 20-30 females per dish) and egg clutches containing F_1 hybrids were collected from them and reared in beakers. F_1 hybrids of each cross were allowed to mate freely, then 110 mated F_1 females were randomly selected to establish each experimental replicate line. Six lines were established each for the following treatments: SD♀xSH♂ heat-selected, SD♀xSH♂ controls, SH♀xSD♂ heat-selected, and SH♀xSD♂ controls. Experimental evolution for thermal tolerance in the 12 heat-selected lines was conducted by exposing each line's beaker (harboring the 110 mated females) to a stress temperature for 1 hr in a pre-heated water bath (starting at 35.7°C for the F_1 generation and increasing by 0.1°C every subsequent generation). Following stress, beakers were returned to incubators and allowed to recover for 1 hr in a 20°C water bath. Three days later, all surviving females were transferred to a new beaker and monitored for the appearance of egg clutches. Between 35 and 50 egg clutches (based on availability per generation) were haphazardly collected from them in order to establish the next generation. Copepods of each new generation were allowed to reach maturity and mate freely before all mated females (125-250 individuals; median = 173) were collected and transferred to a new beaker for exposure to the next stress temperature. The 12 control lines were maintained at 20°C in incubators and new generations were established by haphazardly selecting a number of mated females from each beaker that simulated the average amount of mortality seen in the heat-selected lines of that cross during

that generation, transferring those females to a new beaker, and collecting 50 egg clutches from them. Copepods of each new generation in control lines were reared, allowed to mate, and collected in the same manner as in the heat-selected lines prior to the next sampling. Heat-selected lines were propagated with selection through the F_{10} generation, then the F_{11} generation was cultured without exposure to stress in order to recover the size of each population before F_{12} individuals were reared from eggs and used for final phenotypic assays. Control lines similarly did not experience simulated mortality during F_{11}, and F_{12} individuals were used for assays. During the course of the experiment one SD♀xSH♂ control and two SH♀xSD♂ control replicates experienced population crashes, resulting in 21 total replicates.

Thermal tolerance measurements by LD_{50}

The median lethal temperature (LD_{50}) of each replicate line was measured during the F_4, F_8, and F_{12} generations. Individual male copepods were transferred in 100 µl of ASW to each of 60 single wells in a 96-well PCR plate such that 10 copepods each would be exposed to one of six temperatures in a gradient-enabled thermocycler. Peak stress temperatures ranged from 34.5°C to 37°C in 0.5°C increments, and copepods were exposed to ramping temperatures of 25°C, 28°C, and 32°C for 3 min each prior to being exposed to the peak stress temperature for 1 hr. Following stress, the 96-well plate was placed in a cooling block at 20°C for 30 min to allow for recovery. Copepods were then transferred to individual wells in 24-well plates and fed, and survival of each individual was checked after 3 days. Survival status and temperature were used to estimate the LD_{50} of each assay in R using a binomial linear model followed by the dose function in the MASS package (v7.3-51.4; Venables and Ripley 2002). A two-way ANOVA on the estimated LD_{50} temperatures followed by Tukey *post hoc* and Cohen's *d* effect size tests (effectsize package, v0.6.0.7; Ben-Shachar et al. 2020) was used to test for the effect of experimental group (cross and treatment).

Thermal stability of Complex I-driven ATP synthesis

Thermal stability of ATP synthesis in the F_{12} generation of each line was assessed using a modified protocol from Kayhani and Barreto (2023). For each replicate line, mitochondria were isolated from pools of 24 F_{12} copepods (12 female, 12 male) and assayed *in vitro* for rate of Complex I (CI)-driven ATP synthesis at five temperatures (20, 30, 33, 36, and 39°C), with n = 3 assay pools (72 individuals) per replicate line. Mitochondrial isolation, CI-driven ATP synthesis assays, protein quantification, and rate calculation were conducted following Kayhani and Barreto (2023). Briefly, CI-driven ATP synthesis assays were conducted by combining 25 µl

aliquots of mitochondrial isolates from each replicate with 5 µl of substrate cocktail (ADP, malate, pyruvate, and glutamate) formulated to drive electron donation via OXPHOS CI activity, The reactions were then incubated at the above temperatures simultaneously in a gradient-enabled thermocycler for 15 min. Following this, 25 µl of the incubated isolates were combined with 25 µl of Celltiter-Glo (Promega, Madison, WI, USA) to enable quantification of ATP via luminescence. An additional 25 µl aliquot of each isolate was combined with Celltiter-Glo without incubation to quantify 'initial' ATP concentration in each assay. All isolates were incubated in Celltiter-Glo at room temperature for 10 min prior to being read for luminescence in a Tecan Spark plate reader (Tecan Genomics, Redwood City, CA, USA) alongside ATP standards ranging 3.2 nmol l^{-1} to 10 µmol l^{-1}. Rates were calculated by subtracting the luminescence values of the 'initial' isolates from the incubated isolates and then normalized by protein content of each isolate, which was quantified using NanoOrange Protein Quantification Kit assays (Thermo Fisher Scientific, Waltham, MA, USA) on an additional 5 µl of each isolate. Two-way ANOVAs were used to estimate the effect of temperature on ATP synthesis rate for each experimental group individually in R. Welch's two-sided *t*-tests and Cohen's *d* effect size tests were performed to compare the differences in mean ATP synthesis rates between adjacent temperature treatments within each experimental group.

Life history traits

Following the F_{12} generation, each line was tested for fecundity (number of nauplii hatched from the first clutch), developmental rate (number of days to metamorphosis), viability (proportion alive at day 15 post-hatch), and metamorphosis success (proportion metamorphosed at day 15 post-hatch). For each line, mate pairs were obtained from F_{12} beakers and maintained in petri dishes, which were monitored biweekly for the appearance of red egg clutches on females. Red egg clutches were removed with a needle and transferred to their own well of a 6-well plate and fed with ground dried *Spirulina*. The date of egg-picking was recorded as the hatch date and the number of hatched nauplii was counted 48 hrs later to ensure all eggs had hatched by the time of counting. Clutches that were unhatched after 48 hrs were discarded. Starting on day 5 post-hatch, wells were monitored daily for the appearance of a copepodid (metamorphosed juvenile). When at least one was observed, the date was recorded as the number of days to metamorphosis. On day 15 post-hatch for each well, the total number of living individuals and how many of those had metamorphosed were recorded to determine viability and metamorphosis success for the clutch. Because lines varied in how many mate pairs were available this late in the experiment, not all lines were successfully sampled, with technical

replication ranging from 1-12 per replicate line (median: 4). Linear mixed-effects models were used to test for each life history trait separately, with experimental group as a main factor and line as a random factor in R (nlme package, v3.1.162; Pinheiro et al. 2024).

Pool-seq library preparation and whole-genome sequencing

For each of the 21 replicates during the F_{12} generation, genomic DNA was isolated from pools of 85-150 adult individuals using standard phenol:chloroform protocols (Sambrook and Russell 2006) with the addition of RNase A to reduce RNA contamination. Isolated DNA was quantified using a Qubit fluorometer (Thermo Fisher, Waltham, MA) and diluted such that 25 ng of DNA from each sample were used for subsequent library preparation. Whole-genome libraries were prepared using a modified Nextera protocol (Illumina, San Diego, CA). Briefly, isolated DNA was tagmented using the Nextera tagmentation enzyme (Illumina, San Diego, CA) and then cleaned with a MinElute PCR Purification Kit (Qiagen, Hilden, Germany). Following this, each tagmented DNA sample was tagged with unique, custom-designed dual-index pairs via PCR amplification with the following reaction profile: 3 min at 72°C, 30 sec at 98°C, followed by 11 cycles of 10 sec at 98°C, 30 sec at 63°C, and 3 min at 72°C. Post-PCR samples were quantified again by Qubit fluorometer and 486 ng of DNA from each replicate were pooled and concentrated using a Qiagen MinElute PCR Purification Kit. Following purification, the pool was quantified and diluted to 10nM in 30 µl and sequenced as 150-bp paired-end reads on one S4 flow cell of an Illumina NovaSeq 6000 at the Genomics & Cell Characterization Core Facility (University of Oregon, Eugene, OR).

Read mapping and quantification of SNP frequencies

A pipeline for read mapping and quantification of SNP frequencies was modified from Lima and Willett (2018) and Healy and Burton (2023). Reads from each of the 21 F_{12} samples were trimmed for base quality and adaptor contamination using BBDuk (Bushnell 2014) with default settings. To avoid potential reference bias in mapping caused by using a single reference genome, we mapped reads to a "hybrid" reference genome created to incorporate SD and SH variation equally. The hybrid reference was created by first aligning the SD assembly (Barreto et al. 2018) with the SH assembly (Han and Barreto 2021) and then equalizing the quality of assemblies by making gaps ("N" positions) match between them. Reads were mapped to this hybrid reference using bwa-mem (v0.7.17-r1188; Li 2013) and filtered with samtools (v1.19.2; Danecek et al. 2021) to remove alignments with MAPQ scores less than 20. We used PoPoolation2 (v1201; Kofler et al. 2011) to determine allele counts that mapped to each

parental genome, using a minimum coverage of 50, maximum coverage of 500, and a minimum allele read count of 4. We retained SNP positions that were shared by both parental populations and compared them to a catalog of known fixed SNPs between SD and SH (Han and Barreto 2021). Across the 21 samples, 2,425,402 SNP positions were retained for subsequent statistical analyses.

Since paternal leakage of mtDNA has been reported in certain *T. californicus* hybrid crosses (Lee and Willett 2022), we examined whether this phenomenon may be occurring in our samples. For this, we simply counted reads mapping to each mtDNA chromosome (SD or SH) in the "hybrid" reference (without any further variant calling steps or filters) for each of the 21 hybrid samples. In addition to paternal leakage, other factors can cause mitochondrial reads from one mitotype to map to the alternative mitotype. These include sequencing errors or rare shared polymorphisms. We hence also examined how pure parental data sets behave when mapped to the same hybrid reference. We obtained Pool-seq data for a pure SD and a pure SH from NCBI (SD accessions: SRR27277355 and SRR27277345; SH accession: SRR9019163) and mapped them to the reference to estimate a background level of read mapping to the alternative mitotype.

Statistical modeling and functional annotation

Statistical models were run in R (v4.3.1; R Core Team 2019) using RStudio (v 2023.12.1.402) with the tidyverse package (v2.0.0; Wickham et al. 2019). All figures were generated using ggplot2 (v3.5.0; Wickham 2016) or Integrated Genomics Viewer v2.19.4; (Robinson et al. 2011). Allele counts at each SNP position were used to estimate allele frequencies at those positions for each replicate line. These frequencies were then used to re-scale allele counts such that the adjusted read depth was an arbitrary 50X at all SNPs; variation in sequencing coverage is thought to lead to false positives and re-scaling allele frequencies to counts out of a common value reduces this effect (Wiberg et al. 2017). We then modeled allele frequencies using a generalized linear model with a binomial distribution (binomial GLM), incorporating a likelihood ratio test (*cf.* Wiberg et al. 2017). Model factors included the effects of selection treatment (heat-selected or control), cross direction (SD♀xSH♂ or SH♀xSD♂), and the interaction between them (treatment x cross). This approach allowed us to estimate the effects of selection regime and cross direction on genome-wide allele frequencies between experimental groups, while accounting for their interaction in each case. The 1% of SNPs with the smallest *p*-values (largest $-\log_{10}$ transformed *p*-values) for the effect of treatment were considered regions of the genome likely evolving in response to thermal selection. These SNPs show the most consistent

and significant variation between the four experimental groups in response to the selection treatment, so this comparison represents differences in the extent of introgression based on group. This list of SNP positions was compared to the annotated SD reference genome (Barreto et al. 2018) using bedtools intersect (Quinlan and Hall 2010) to categorize SNPs as intergenic, exonic, or intronic, and to identify which genes populated those genomic regions. If a SNP overlapped with two genes that are transcribed on opposite strands, both genes were retained in the final list. Functional annotations of these genes were then mined to test for enrichment of cellular functions based on gene ontology (GO) terminology with topGO (v2.54.0; Alexa and Rahnenfuhrer 2024). We also tested for overrepresentation of certain categories of interest in this system: (i) nuclear-encoded mitochondrially targeted proteins (MTPs); (ii) mtDNA-interacting MTPs; and (iii) heat shock protein genes (*HSPs*). The first two categories were compiled by Barreto et al. (2018), while the last was compiled in this study by identifying genes annotated as being from any *HSP* gene family. Additionally, the 1% of SNPs with the smallest *p*-values for the effect of cross direction were considered regions of the genome evolving in response to differences between reciprocal crosses outside of thermal adaptation, suggesting that selection for favorable mitonuclear combinations may have driven allele frequency variation in these regions. Genes housing these SNPs were identified as described previously and mined for functional annotations.

Assessment of statistical threshold choice

As described in the main text, we chose to use a 1% genome-wide statistical threshold to prioritize regions of highest impact, as this threshold is more stringent than the widely used Bonferroni correction. The latter approached would have retained over eight times as many SNP loci (209,864, **Supplementary Fig. S5**). To assess whether the patterns of allele frequency are affected by the choice of threshold, we briefly examined the SNP frequencies across chromosomes using the Bonferroni threshold and compared them to our complete analysis at 1%.

We found that the larger set of SNPs assessed by the Bonferroni approach clearly recapitulates the trends captured by our original 1% cutoff. This can be seen within chromosomes. For instance, Chromosome 2 had over 5x more SNPs (N = 106,898) with Bonferroni than with our original cutoff (N = 23,133) but showed the same pattern (**Supplementary Fig. S2 and S6**). Indeed, this concordance was observed for all six chromosomes highlighted with our 1% threshold (in **Supplementary Fig. S2**), indicating that our original more stringent statistical cutoff did not miss any chromosome-wide patterns.

Further, the Bonferroni cutoff is low enough to capture significant SNP loci from every chromosome, so the patterns in Chromosomes 1, 4, 5, 8, 9, and 11 (beyond the 1 SNP found on Chromosome 11 originally) can be examined for the first time here. Overall, these match the genome-wide pattern. Chromosomes 1, 5, and 11 match strongly, while 4 and 8 match weakly and chromosome 9 shows a slight difference but it is also weak. This variation in the strength of the patterns is likely due to the wide range of number and their significance level of loci among chromosomes. For instance, Chromosomes 4 and 9, with weak patterns, have few SNPs (73 and 56, **Supplementary Fig. S6**) and these SNPs are only mildly above the Bonferroni threshold (**Supplementary Fig. S5**).

We conclude that the more permissive Bonferroni cutoff would not change any of our primary results regarding the allele frequency response of the different treatments. The Bonferroni approach would of course result in a much larger list of genes to be included in downstream analyses. This larger list comes with the trade-off of potentially finding more implicated genes but also including irrelevant genes of very weak or no effect. We favor being cautious when discussing potential genes and functions. By focusing on the highest regions of allele frequency difference (via the stringent 1% cutoff), we will emphasize variants of highest relevance.

References

Alexa A, Rahnenfuhrer J. 2024. topGO: Enrichment Analysis for Gene Ontology. Bioconductor doi:10.18129/B9.bioc.topGO, R package version 2.54.0, https://bioconductor.org/packages/topGO

Barreto FS, Watson ET, Lima TG, Willett CS, Edmands S, Li W, Burton RS. 2018. Genomic signatures of mitonuclear coevolution across populations of Tigriopus californicus. *Nat Ecol Evol.* 2:1250–1257. https://doi.org/10.1038/s41559-018-0588-1

Ben-Shachar MS, Lüdecke D, Makowski D. 2020. effectsize: Estimation of effect size indices and standardized parameters. *J. Open Source Softw.* 5:2815. https://doi.org/10.21105/joss.02815

Bushnell B. 2014. BBTools. *DOE Joint Genome Institute* [Internet]. https://jgi.doe.gov/data-and-tools/software-tools/bbtools/

Danecek P, Bonfield JK, Liddle J, Marshall J, Ohan V, Pollard MO, Whitwham A, Keane T, McCarthy SA, Davies RM, et al. 2021. Twelve years of SAMtools and BCFtools. *GigaScience* 10:giab008. https://doi.org/10.1093/gigascience/giab008

Han K-L, Barreto FS. 2021. Pervasive mitonuclear coadaptation underlies fast development in interpopulation hybrids of a marine crustacean. *Genome Biol Evol*. 38: evab004 https://doi.org/10.1093/gbe/evab004

Healy TM, Burton RS. 2023. Differential gene expression and mitonuclear incompatibilities in fast- and slow-developing interpopulation *Tigriopus californicus* hybrids. *Mol Ecol.* 32:3102–3117. https://doi.org/10.1111/mec.16917

Kayhani K, Barreto FS. 2023. Disproportionate role of nuclear-encoded proteins in organismal and mitochondrial thermal performance in a copepod. *J Exp Biol*. 226:jeb246085. https://doi.org/10.1242/jeb.246085

Kofler R, Pandey RV, Schlötterer C. 2011. PoPoolation2: identifying differentiation between populations using sequencing of pooled DNA samples (Pool-Seq). *Bioinformatics.* 27:3435–3436. https://doi.org/10.1093/bioinformatics/btr589

Lee J, Willett CS. 2022. Frequent paternal mitochondrial inheritance and rapid haplotype frequency shifts in copepod hybrids. *J Hered* 113:171–183. https://doi.org/10.1093/jhered/esab068

Li H. 2013. Aligning sequence reads, clone sequences and assembly contigs with BWA-MEM. http://arxiv.org/abs/1303.3997

Lima TG, Willett CS. 2018. Using pool-seq to search for genomic regions affected by hybrid inviability in the copepod *T. californicus*. *J Hered.* 109:469–476. https://doi.org/10.1093/jhered/esx115

Pinheiro J, Bates D, DebRoy S, Sarkar D, Heisterkamp S, Van Willigen B, Ranke J, R Core Team. 2024. nlme: Linear and nonlinear mixed effects models. https://cran.r-project.org/web/packages/nlme/index.html

Quinlan AR, Hall IM. 2010. BEDTools: a flexible suite of utilities for comparing genomic features. *Bioinformatics.* 26:841–842. https://doi.org/10.1093/bioinformatics/btq033

R Core Team. 2019. R: A language and environment for statistical computing. https://www.R-project.org/

Robinson JT, Thorvaldsdóttir H, Winckler W, Guttman M, Lander ES, Getz G, Mesirov JP. 2011. Integrative Genomics Viewer. *Nat Biotechnol.* 29:24–26.

Sambrook J, Russell DW. 2006. Purification of Nucleic Acids by Extraction with Phenol:Chloroform. *Cold Spring Harb Protoc* 2006:pdb.prot4455. http://cshprotocols.cshlp.org/content/2006/1/pdb.prot4455

Venables WN, Ripley BD. 2002. Modern Applied Statistics with S. Fourth. New York: Springer http://www.stats.ox.ac.uk/pub/MASS4

Wiberg RAW, Gaggiotti OE, Morrissey MB, Ritchie MG. 2017. Identifying consistent allele frequency differences in studies of stratified populations. *Methods in Ecology and Evolution* 8:1899–1909. https://doi.org/10.1111/2041-210X.12810

Wickham H. 2016. ggplot2: Elegant Graphics for Data Analysis. Springer-Verlag New York https://ggplot2.tidyverse.org

Wickham H, Averick M, Bryan J, Chang W, McGowan LD, François R, Grolemund G, Hayes A, Henry L, Hester J, et al. 2019. Welcome to the tidyverse. *J. Open Source Softw.* 4:1686.

Supplementary Table S1. *Post hoc* estimates of difference in LD_{50} by experimental group. Shown are *P*-values from Tukey multiple comparisons tests (α = 0.05) and Cohen's *d* effect size test statistics for each comparison.

Comparison	Difference (°C)	Adjusted *P*-value	*d*
SD♀xSH♂ selected - SD♀xSH♂ control	0.459	0.014	0.94
SH♀xSD♂ selected - SH♀xSD♂ control	0.674	2.04×10^{-4}	1.71
SH♀xSD♂ selected - SD♀xSH♂ selected	0.113	0.868	0.35
SH♀xSD♂ control - SD♀xSH♂ control	-0.101	0.909	0.19

Supplementary Table S2. Changes in complex I-driven ATP synthesis rates between adjacent assay temperatures in each of four experimental groups. Shown are *P*-values from Welch's two-sided *t* tests (α = 0.05) and Cohen's *d* effect size test statistics for each comparison. Analyses were performed for each experimental group independently.

Comparison	*P*-value	Cohen's *d*
SD♀xSH♂ selected		
20°C to 30°C	0.005	1.00
30°C to 33°C	0.915	0.04
33°C to 36°C	0.266	0.38
36°C to 39°C	4.93×10^{-5}	1.63
SD♀xSH♂ controls		
20°C to 30°C	0.005	0.78
30°C to 33°C	0.585	0.20
33°C to 36°C	0.213	0.47
36°C to 39°C	5.54×10^{-6}	2.23
SH♀xSD♂ selected		
20°C to 30°C	0.045	0.69
30°C to 33°C	0.291	0.36
33°C to 36°C	0.019	0.82
36°C to 39°C	1.43×10^{-9}	2.99
SH♀xSD♂ controls		
20°C to 30°C	0.245	0.49
30°C to 33°C	0.827	0.09
33°C to 36°C	0.430	0.33
36°C to 39°C	4.10×10^{-4}	1.82

Supplementary Table S3. Read mapping statistics across the 21 sequenced pools of individuals at generation F_{12}.

Replicate line	Mean read depth (genome-wide)	Range of mean depth (across chromosome)	Mean % Coverage (genome-wide)	Range of % coverage (across chromosomes)
SD♀xSH♂ Selected 1	152x	139x-176x	83.75	70.15-87.48
SD♀xSH♂ Selected 2	180x	166x-207x	83.82	70.29-87.50
SD♀xSH♂ Selected 3	242x	227x-268x	83.89	70.44-87.56
SD♀xSH♂ Selected 4	157x	145x-181x	83.76	70.20-87.48
SD♀xSH♂ Selected 5	129x	118x-148x	83.75	70.21-87.45
SD♀xSH♂ Selected 6	79x	73x-93x	83.63	70.05-87.38
SD♀xSH♂ Control 1	120x	108x-138x	83.70	70.13-87.42
SD♀xSH♂ Control 3	169x	156x-191x	83.72	70.11-87.45
SD♀xSH♂ Control 4	160x	146x-182x	83.67	70.07-87.44
SD♀xSH♂ Control 5	157x	148x-182x	83.78	70.15-87.48
SD♀xSH♂ Control 6	106x	99x-125x	83.65	70.02-87.38
SH♀xSD♂ Selected 1	104x	95x-116x	83.61	70.11-87.40
SH♀xSD♂ Selected 2	81x	74x-96x	83.61	69.92-87.33
SH♀xSD♂ Selected 3	136x	123x-156x	83.71	70.10-87.42
SH♀xSD♂ Selected 4	220x	200x-251x	83.87	70.39-87.56
SH♀xSD♂ Selected 5	175x	158x-197x	83.76	70.11-87.46
SH♀xSD♂ Selected 6	220x	206x-243x	83.84	70.39-87.55
SH♀xSD♂ Control 1	159x	147x-177x	83.73	70.13-87.45
SH♀xSD♂ Control 4	144x	133x-162x	83.72	70.13-87.42
SH♀xSD♂ Control 5	157x	146x-175x	83.76	70.24-87.48
SH♀xSD♂ Control 6	169x	158x-190x	83.80	70.20-87.52

Supplementary Table S4. Test of mtDNA paternal leakage. Shown are counts of reads mapped to a "hybrid assembly" containing SD and SH mitochondrial chromosomes. The "incorrect" mtDNA refers to the reference mitotype that is alternative to the maternal background in each sample.

Sample	Reads mapped to SD mtDNA	Reads mapped to SH mtDNA	% mapped to incorrect mtDNA
Pure SD[1]	442,608	274	0.062%
Pure SH[2]	4,042	1,820,658	0.222%
SD♀xSH♂ Control 1	460,495	3,962	0.853%
SD♀xSH♂ Control 3	582,340	157	0.027%
SD♀xSH♂ Control 4	574,718	982	0.171%
SD♀xSH♂ Control 5	583,812	122	0.021%
SD♀xSH♂ Control 6	370,861	67	0.018%
SD♀xSH♂ Selected 1	583,423	1,568	0.268%
SD♀xSH♂ Selected 2	628,441	1,100	0.175%
SD♀xSH♂ Selected 3	900,512	5,107	0.564%
SD♀xSH♂ Selected 4	591,001	2,013	0.339%
SD♀xSH♂ Selected 5	526,801	457	0.087%
SD♀xSH♂ Selected 6	312,514	584	0.187%
SH♀xSD♂ Control 1	1,076	537,237	0.200%
SH♀xSD♂ Control 4	888	489,177	0.181%
SH♀xSD♂ Control 5	874	558,001	0.156%
SH♀xSD♂ Control 6	1,410	651,047	0.216%
SH♀xSD♂ Selected 1	646	403,200	0.160%
SH♀xSD♂ Selected 2	387	235,100	0.164%
SH♀xSD♂ Selected 3	2,014	512,447	0.391%
SH♀xSD♂ Selected 4	541	650,504	0.083%
SH♀xSD♂ Selected 5	2,547	865,002	0.294%
SH♀xSD♂ Selected 6	1,781	921,554	0.193%

[1]The SD sample was a pool of two pure samples from NCBI (accessions: SRR27277355 and SRR27277345), totaling ~110 million reads.

[2]The SH sample was from Han and Barreto (2021) (accession SRR9019163) and contained ~253 million reads.

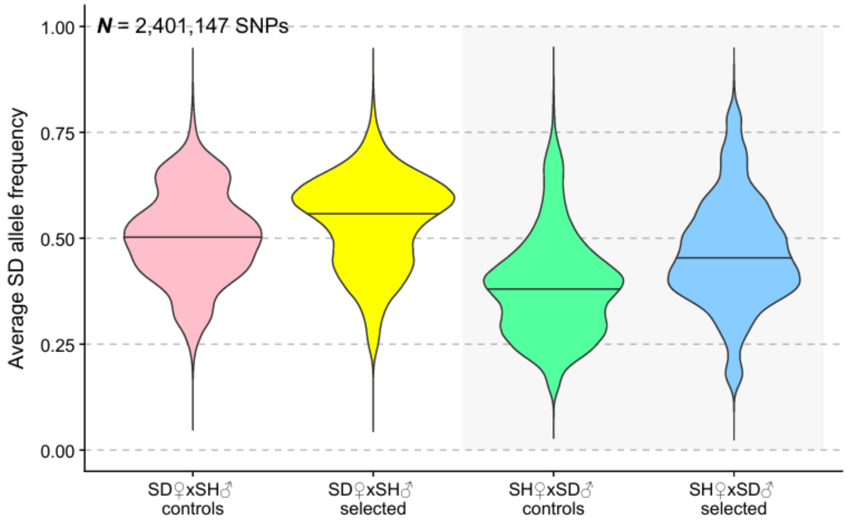

Supplementary Figure S1. Average SD allele frequency in SNPs found not to be significant for selection treatment effect (heat-selection vs control regime). Data shown are calculated from 2,401,147 genome-wide SNPs that did not pass statistical threshold (see Figure 1 in main text). Averages are estimated across replicate lines in each group. Horizontal lines in violin plots mark the median of the distributions.

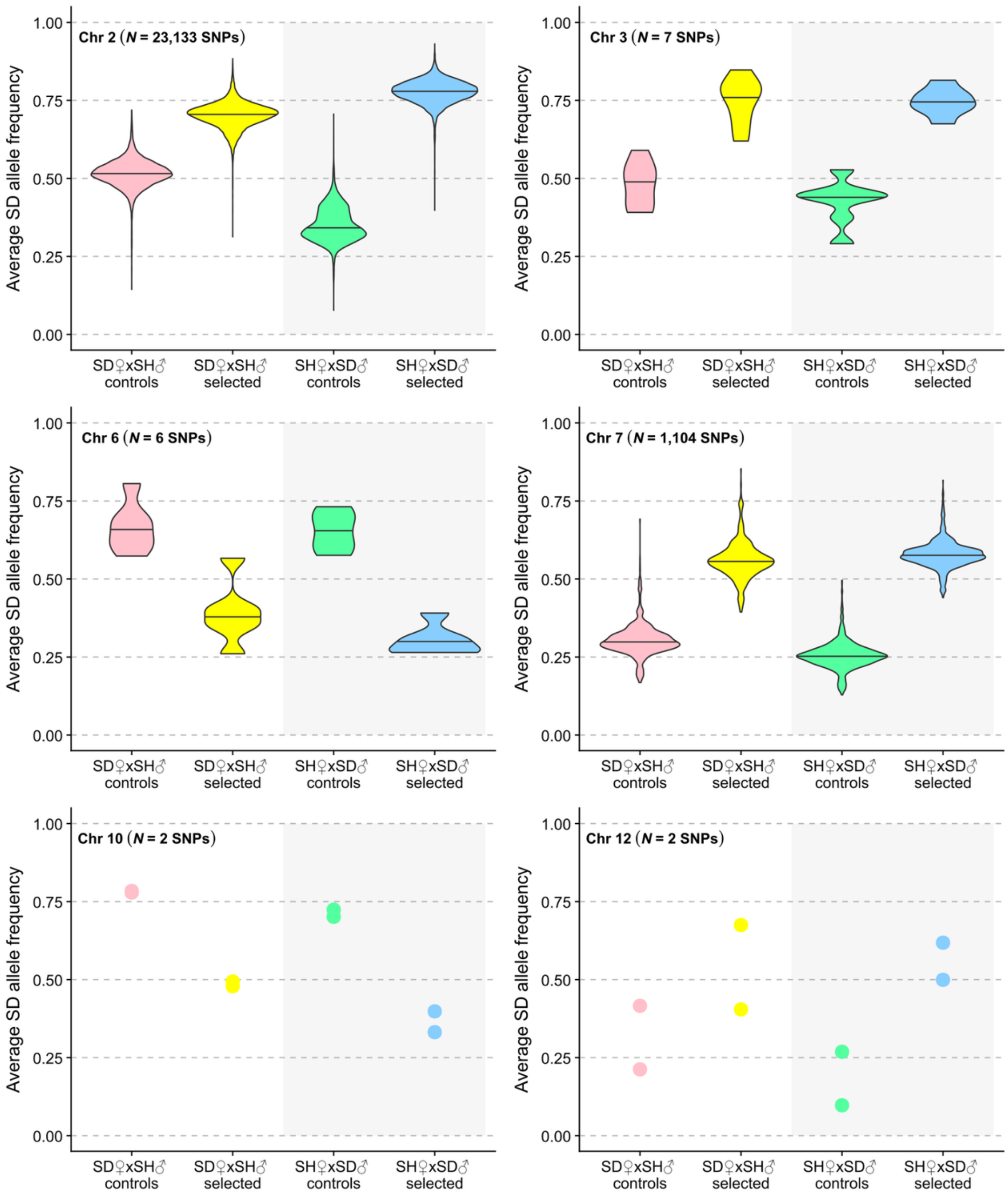

Supplementary Figure S2. Average SD allele frequency in SNPs with strong signal of treatment effect (heat-selection vs control regime). Data shown are from six chromosomes containing more than one SNP locus passing statistical threshold (see Figure 1 in main text). Averages are estimated across replicate lines in each group. Horizontal lines in violin plots mark the median of the distributions. Chromosomes with only 2 significant loci have values as points. Chromosome 11 is not included in this plot as it contained only one significant SNP.

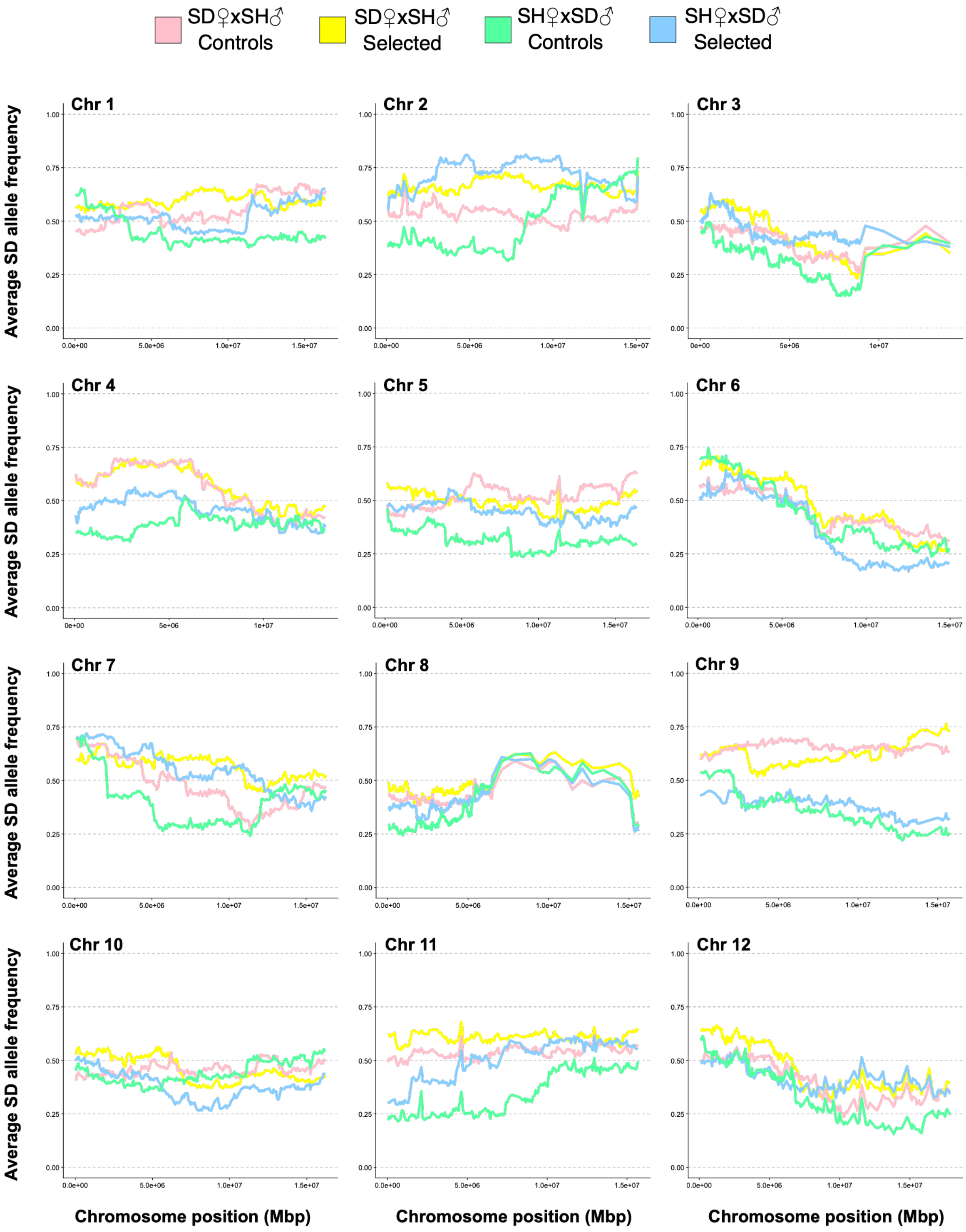

Supplementary Figure S3. Average SD allele frequency of each experimental group along each chromosome. Plots show the mean of within-group SD allele frequencies, and averaged across loci in 200 non-overlapping windows on each chromosome.

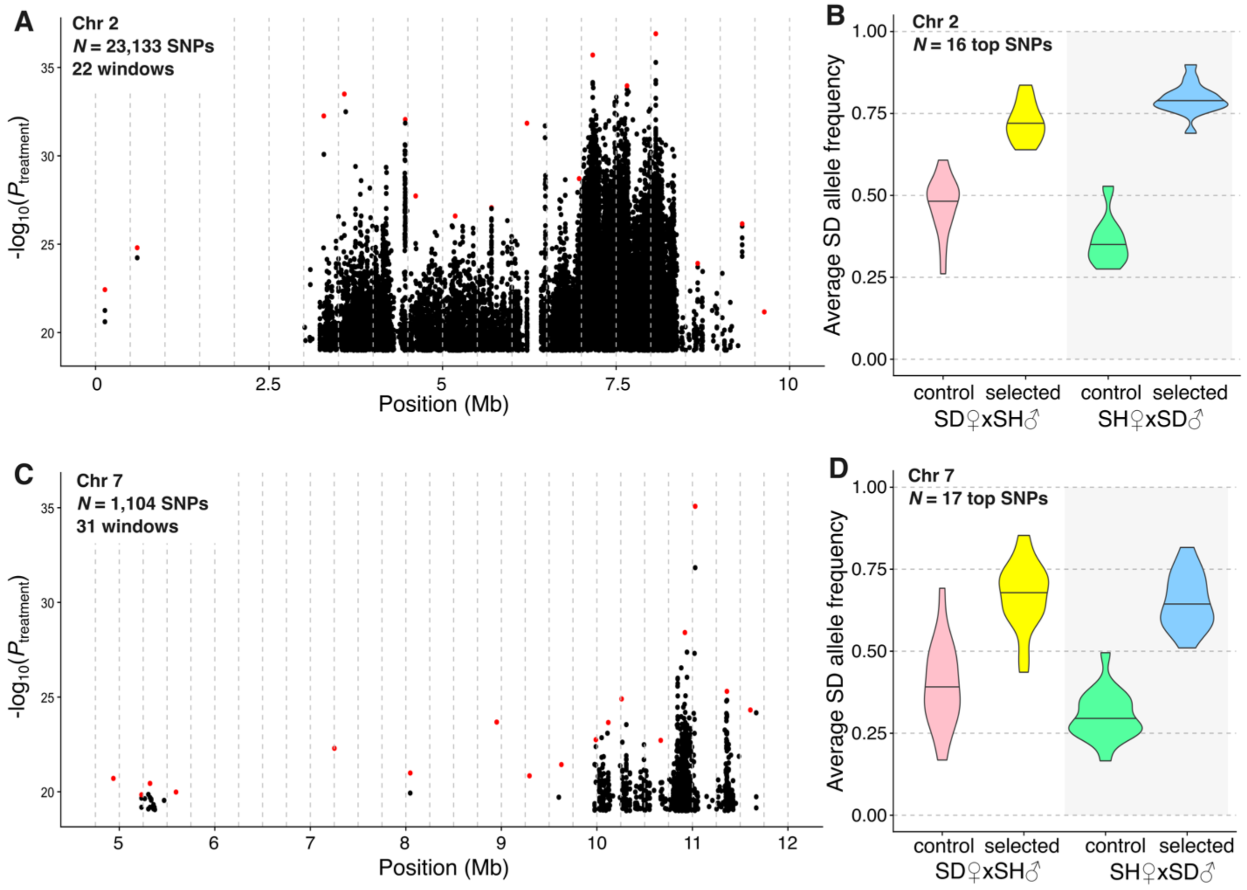

Supplementary Figure S4. Windowing analysis of chromosomes 2 and 7 of SNP loci with significant effect of selection regime (heat-selection vs control). Data are for the chromosomes with strongest signal. (**A** and **C**) Points shown are of all SNP loci above statistical threshold, and vertical dashed lines mark boundaries of 500-kb (**A**) and 250-kb (**C**) windows. Red points mark the most significant SNP per window, which were used to calculate new averages (**B** and **D**).

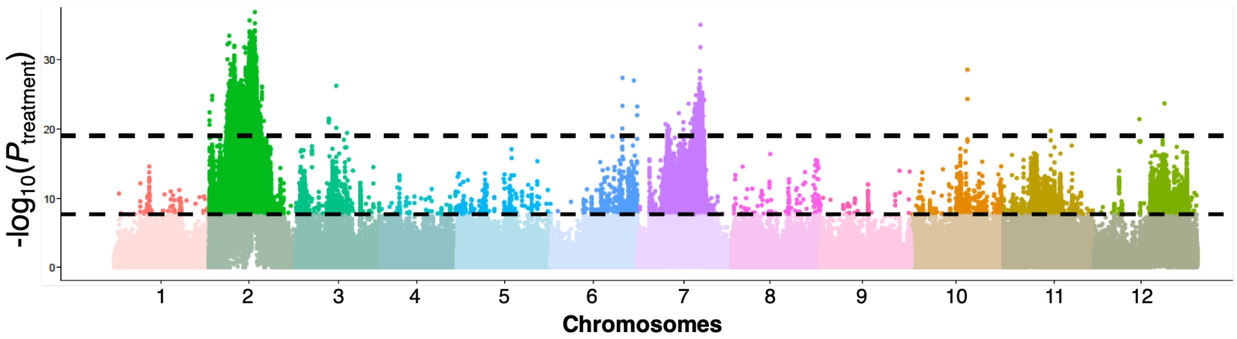

Supplementary Figure S5. Manhattan plot of statistical tests for the effect of thermal selection treatment assessed at two genome-wide thresholds. Plotted are re-scaled *P*-values for all SNP loci (N = 2,425,402). The top horizontal dashed line marks the threshold of the 1% most significant SNPs (*N* = 24,255 SNPs; *P* = 1.01×10^{-19}). The bottom dashed line marks the Bonferroni threshold (*P* = 2.06×10^{-08})

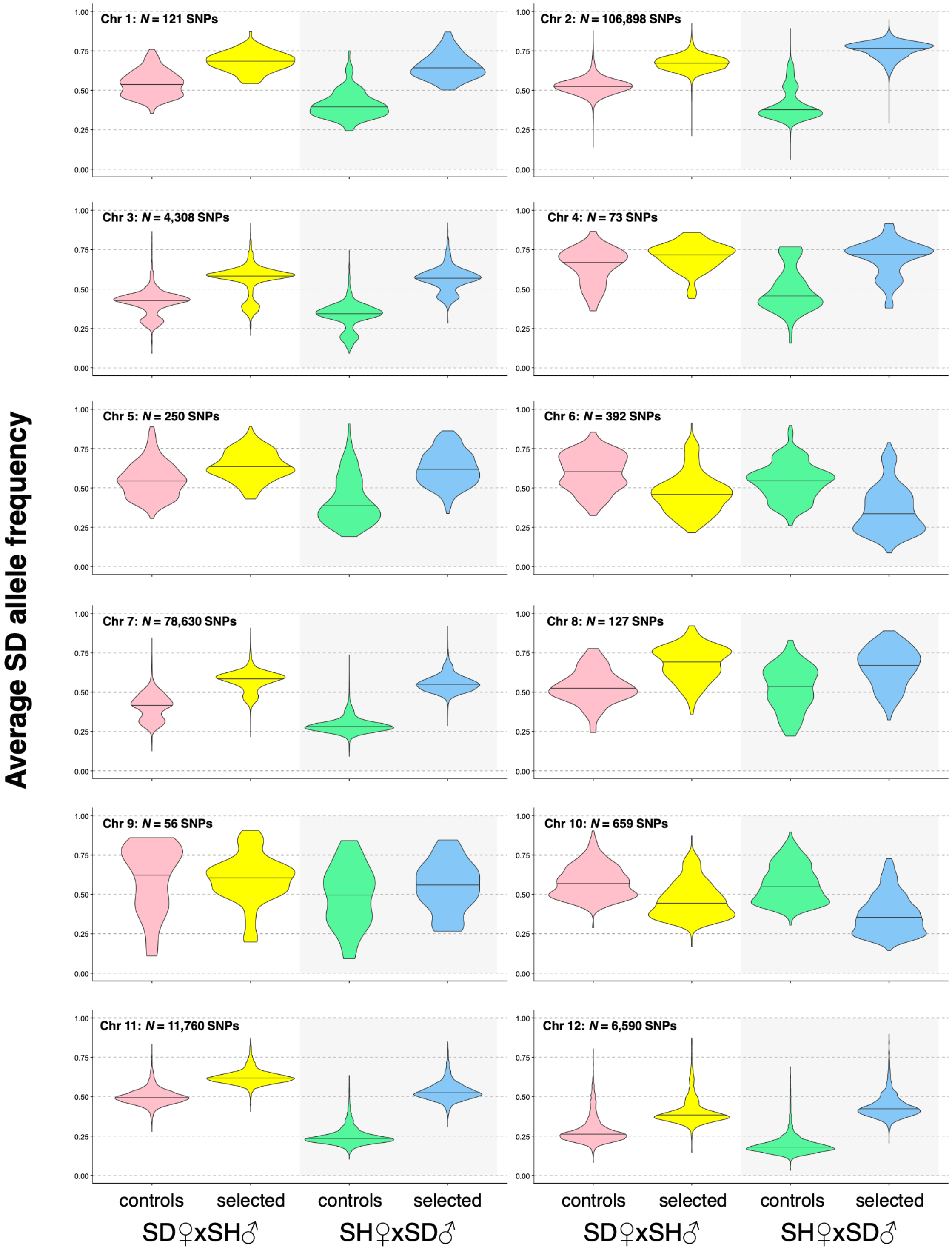

Supplementary Figure S6. Violin distribution plots of average SD allele frequency in SNPs significant for treatment effect (heat-selection vs control regime) at the Bonferroni threshold. This figure is similar to Figure S2 above but instead incorporates loci significant at a lower genome-wide threshold following the Bonferroni method (as per Figure S5).

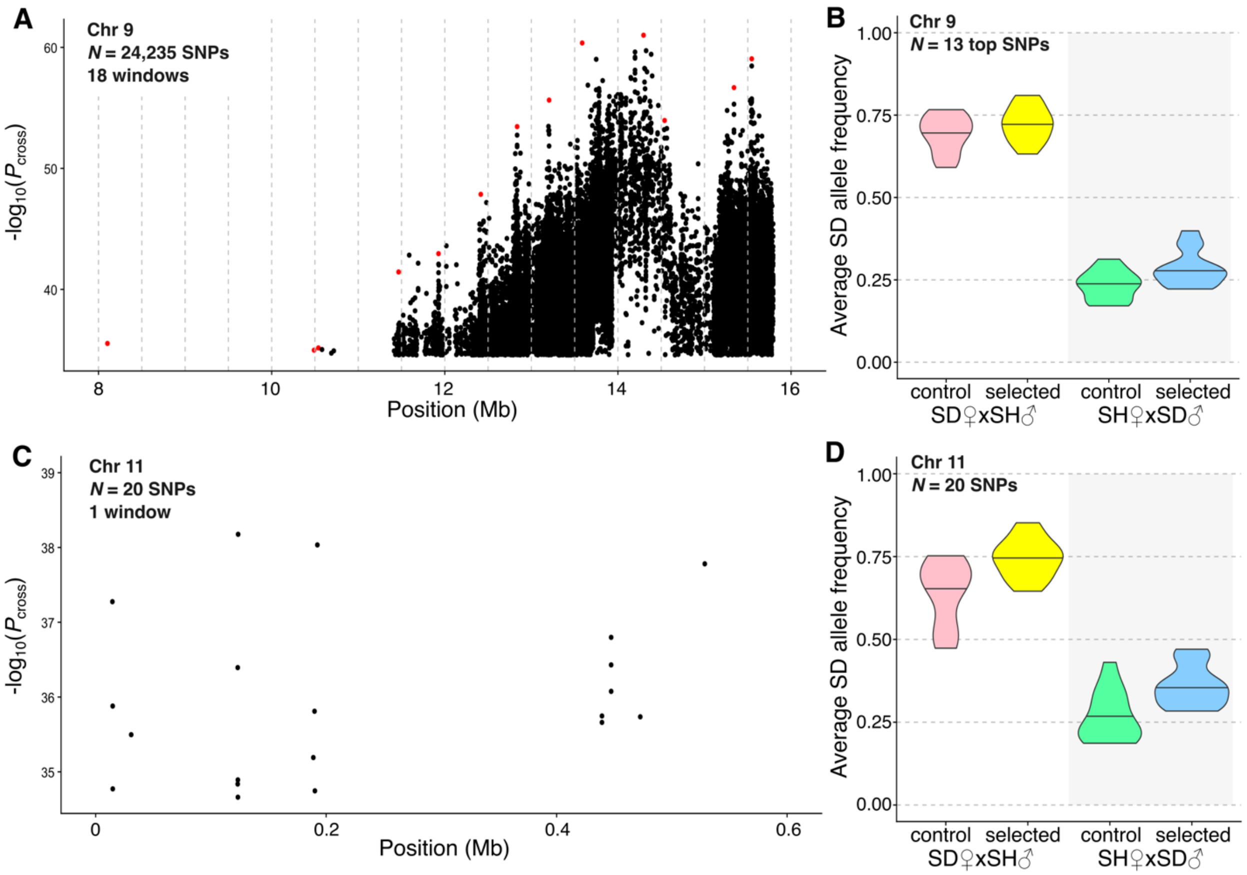

Supplementary Figure S7. Windowing analysis of chromosomes 9 and 11 of SNP loci with significant effect of cross direction. Data are for the chromosomes with strongest signal. (**A**) Points show are of all SNP loci above statistical threshold, and vertical dashed lines mark boundaries of 500-kb windows. Red points mark the most significant SNP per window, which were used to calculate new averages (**B**). (**C**) All significant SNP loci in chromosome 11. No windowing was performed because of the small region, so all loci were used for estimating averages (**D**).
